# Energy-conscious scheduling in edge environments: hybridization of traditional control and DE algorithm

**DOI:** 10.3389/frobt.2025.1656516

**Published:** 2025-12-05

**Authors:** Kun Ma, Lingyu Xu

**Affiliations:** Thanh Dong University, Hai Phong City, Vietnam

**Keywords:** edge computing, task scheduling, energy-saving optimization, hybrid differential evolution algorithm, edg eresource management

## Abstract

Robot applications encompass a multitude of edge computing tasks, such as image processing, health monitoring, path planning, and infotainment. However, task scheduling within such environments remains a significant challenge due to the inherent limitations of edge computing resources and the dynamically fluctuating nature of workloads. EdgeCloudSim, a widely used simulation platform for edge computing, supports a conventional control strategy—Least-Loaded First-Fit Decreasing (LLFFD)—that is favored for its simplicity and speed, especially in scenarios with relatively small-scale and stable workloads. However, as the number of tasks grows and task-VM matching becomes more complex, traditional heuristics struggle to optimize resource utilization and energy consumption effectively. To address this, we propose a hybrid scheduling approach—FFDDE—that integrates the FFD heuristic with the Differential Evolution (DE) algorithm for optimized task-to-VM mapping in edge environments. Using the EdgeCloudSim simulation framework, we evaluate both strategies under diverse workload conditions, comparing their performance in terms of energy consumption and task completion time. Experimental results demonstrate that, compared with the traditional LLFFD method and the classic heuristic algorithm—GA, the hybrid DE-based strategy achieves significantly improved energy efficiency through better task consolidation. This study highlights the potential of combining fast heuristic methods with evolutionary optimization to achieve more sustainable task scheduling in edge computing scenarios.

## 1 Introduction

Mobile and edge computing systems must intelligently schedule tasks from user devices onto nearby edge servers to meet latency and efficiency goals. Task scheduling in such distributed edge environments is a complex NP-hard problem with multiple objectives (e.g., response time, energy, resource utilization) Abdel-Basset et al. (2022). Traditional scheduling algorithms (e.g., First-Come First-Served, Round-Robin, greedy heuristics) are fast and simple, but they often yield suboptimal resource usage and energy performance in complex edge scenarios Avan et al. (2023). For example, simple heuristic strategies can schedule tasks quickly, yet they cannot guarantee optimal results and may get stuck in locally optimal configurations, especially under dynamic, multi-factor conditions Wang et al. (2020). Improving resource utilization is critical, since unused capacity wastes energy with no benefit Llorens-Carrodeguas et al. (2021). In cloud and fog computing contexts, many approaches focus on energy-efficient scheduling, but most are evaluated via simulations due to difficulty of real-world testing Tang et al. (2024). Edge computing inherits these challenges and adds constraints like device mobility and limited server power, making energy-conscious task placement even more crucial.

### 1.1 Energy consumption and scheduling

Each active edge server or VM consumes a baseline power even if underutilized. Thus, scheduling algorithms that consolidate workload (i.e., increase average CPU utilization) can reduce the total number of active servers or active time, lowering energy consumption Ahmed et al. (2021). Ullah works have shown that minimizing the number of VMs used and reducing task makespan leads to significant energy savings in fog and cloud environments Ullah et al. (2024). For example, Singh et al. achieved lower energy usage by scheduling workflows such that fewer VMs were active and tasks finished sooner Singh and Kumar (2023). Similarly, Saidi et al.demonstrated that improving VM CPU utilization (through better task allocation) directly reduces overall energy consumption in a cloud data center Saidi and Bardou (2023). These studies underscore that how tasks are matched to resources can markedly impact energy efficiency. Energy-aware scheduling has therefore become a prominent research topic in edge and cloud computing Kocot et al. (2023), with solutions ranging from heuristics to advanced machine learning.

### 1.2 Heuristics vs. metaheuristics

Greedy heuristics like First-Fit Decreasing (FFD) are popular for task scheduling and bin packing due to their simplicity and speed. In FFD, tasks are sorted by decreasing size and then placed onto the first available resource (VM) that can accommodate them. This strategy is easy to implement and often yields an acceptable solution quicklyda da Costa et al. (2023). FFD and related heuristics have beenapplied to cloud/edge scheduling, for instance to allocate tasks to VMs based on workload sizes and VM capacities Chen et al. (2023). However, heuristic methods do not guarantee optimal or near-optimal solutions and can perform poorly for complex multi-dimensional scheduling constraints. Greedy FFD approaches may be less efficient for complex scenarios such as cloud and fog computing Juan et al. (2023). In dynamic edge environments with many variables, heuristics risk getting trapped in local optima and may not adapt well to changing conditions.

To improve upon static heuristics, researchers have turned to metaheuristic and evolutionary algorithms that explore the solution space more broadly. Differential Evolution (DE) is one such evolutionary optimization algorithm known for fast convergence and simplicity of operations Song et al. (2023). DE evolves a population of candidate solutions (scheduling assignments, in our context) by iteratively applying mutation and crossover, and selecting the fittest solutions. It has shown success in many scheduling and resource allocation problems, from cloud task scheduling to IoT edge clustering. Metaheuristics like DE can approach near-optimal solutions for NP-hard scheduling problems, though they involve more computation time than heuristics Ahmad et al. (2022), and Laili et al. (2023). Recent studies have hybridized heuristics with metaheuristics to get the best of both–using heuristics to guide or initialize the search, then applying global optimization to fine-tune the allocation. For example, Abdel-Basset et al. (2022) introduced a hybrid DE (HDE) algorithm with custom improvements to solve cloud task scheduling, achieving better results than both classical DE and simpler schedulers like first come first served algorithm and Round-Robin algorithmAbdel-Basset et al. (2022). Likewise, Chhabra et al. (2022) combined Whale Optimization with oppositional DE to minimize makespan and energy for scheduling independent cloud tasks, and reported 3%–19% reductions in energy use compared to baseline algorithms Chhabra et al. (2022). These works illustrate the promise of hybrid approaches in balancing exploration (global search for energy/timing optimization) with exploitation (greedy efficient packing of tasks). To clearly present and compare the current state of research in edge computing task scheduling, we have analyzed and summarized prominent related works in terms of their main contributions, methods, and limitations in Table 1.

**TABLE 1:** The related works of task scheduling in edge computing.

References	Main contribution	Method	Limitations
	Main contribution & objective		Limitations & research gap
Edge computing empowered digital twin: an end-to-end computing task scheduling approach. Zhong et al. (2024)	Systematically classifies edge computing task scheduling algorithms by integrating attention mechanisms and digital twin technology, aiming to address the challenges of limited communication and computing resources	They developed a deep reinforcement learning (DRL) based approach for-computing task scheduling, named HAT-DRL, which aims to minimize the total completion time for various digital twins (DTs)	This DRL-based approach requires significant training. FFDDE combines a fast heuristic with an evolutionary algorithm, offering energy optimization without extensive training
Joint resource trading and task scheduling in edge-cloud computing networks. Ma et al. (2025)	The paper proposes a framework that enhances resource utilization through effective scheduling and trading mechanisms in edge-cloud computing networks	Multi-round proposer-voter algorithm for homogeneous workloads. Gibbs sampling and distributed alternating update algorithms for heterogeneous workloads	This work focuses on resource trading across a broad edge-cloud network. Our FFDDE specifically addresses the fine-grained, energy-focused task-to-VM mapping problem in a single edge environment
Task scheduling method for edge computing in intelligent building system. Yi et al. (2021)	Edge computing enhances task scheduling by processing data closer to the source, reducing latency. The proposed SSA-GA hybrid scheduling algorithm optimizes task completion time, communication time, and CPU energy consumption	Mathematical model establishment considering communication time, task completion, CPU energy. SSA-GA hybrid scheduling algorithm proposed for optimization efficiency	This paper uses a different hybrid (SSA-GA). Our FFDDE uniquely embeds the FFD heuristic into DE’s initialization and repair, providing a more robust search for energy-optimal solutions
An adaptive mechanism for dynamically collaborative computing power and task scheduling in edge environment. Xu et al. (2021)	Edge computing enhances task scheduling by utilizing local computing, storage, and network resources to efficiently manage user requests. The proposed ADCS mechanism dynamically schedules tasks and computing power, minimizing deadline misses and average completion times in edge environments	Greedy decision method for scheduling computing tasks and Bayes method for adjusting computing resources based on user requests	Their method minimizes deadlines but does not prioritize energy efficiency. FFDDE is explicitly designed to minimize energy consumption by consolidating workloads, filling a key gap
FPGA-based edge computing framework: modeling of computation task scheduling. Tan et al. (2023)	The paper proposes a dynamic task scheduling model for FPGA-based edge nodes, balancing load and improving performance by offloading tasks based on node states and task information	The paper proposes a modeling method for task scheduling within an FPGA-based edge computing framework. It utilizes dynamic task scheduling to achieve load balancing in the edge computing network	This framework is limited to FPGA-based systems. Our FFDDE is a software-based solution for general VM environments, making it more broadly applicable and scalable without specialized hardware

### 1.3 Contribution

This paper proposed a hybrid scheduling strategy—FFDDE—that integrates the traditional First-Fit Decreasing (FFD) heuristic into the Differential Evolution (DE) algorithm for task allocation in edge computing environments. The proposed FFDDE approach is evaluated in comparison to the conventional load-aware FFD (LLFFD) and GA (genetic algorithm), with a focus on energy consumption and task completion time. To the best of our knowledge, this work is among the first to explicitly combine heuristic control methods with evolutionary algorithms in edge computing and quantitatively assess their impact on energy efficiency. Simulations were conducted using Edge CloudSim Nandhakumar et al. (2024), a platform specifically designed for edge computing scenarios, incorporating realistic models of wireless latency, user mobility, and server energy consumption based on CPU utilization. This research demonstrates that embedding domain-specific heuristic knowledge into evolutionary algorithms can lead to more energy-efficient task scheduling without compromising system performance.

## 2 Edge task scheduling model

**FIGURE 1:**
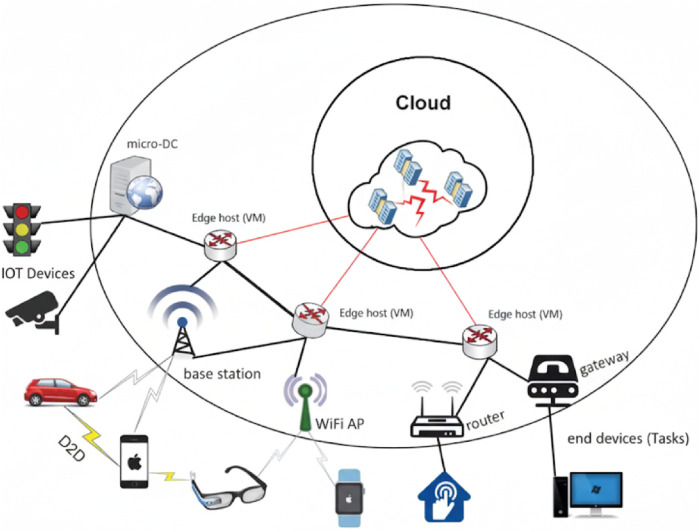
Edge computing model Architecture.

The edge computing model is shown in Figure 1. The most peripheral devices collect information and send corresponding tasks. The edge host of the middle layer processes tasks through VMs. Cloud is the final data center. In order to conduct targeted simulations, this paper makes some settings based on this model (refer to Figure 3 for specific details).

In order to focus on the task allocation problem in the model, this paper assumes that tasks are independent (i.e., there are no dependencies among them) and can be executed on any available edge virtual machine (VM). For instance, a VM with 1000 MIPS can process 1,000 million instructions of computational work per second. To emulate the diversity of real-world edge computing environments, we consider a heterogeneous infrastructure composed of mixed VM types—some with higher processing capacities and others with more limited capabilities.

Each VM is capable of handling multiple tasks concurrently. However, the number of VMs hosted on each edge server is restricted by hardware and resource limitations. Consequently, the task allocation process must comply with these per-node capacity constraints to avoid over-provisioning and ensure system stability. This limitation introduces additional complexity to the scheduling problem, requiring intelligent strategies to achieve efficient task-to-VM mappings under constrained resources. The definitions and descriptions are as follows:

Unified Problem Definition.

Let the task set be $T={\left\{\tau_{i}\right\}}_{i=1}^{N}$ and the virtual machine set be $V={\left\{v_{j}\right\}}_{j=1}^{M}$ , where:

-
$d\left(\tau_{i}\right)$ : The computational demand of task $\tau_{i}$

-
$c\left(v_{j}\right)$ : The computational capacity of virtual machine $v_{j}$

-
$U_{j}\left(t\right)$ : The real-time utilization rate of virtual machine $v_{j}$ at time t $\left(U_{j}\left(t\right)\in \left[0,1\right]\right)$

-
$T_{\mathrm{makespan}}=\max_{\tau_{i}}C\left(\tau_{i}\right)$ : The completion time of task T

### 2.1 Strategy 1- traditional control (LLFFD)

#### 2.1.1 Decision rules

In the baseline scheduling strategy, task allocation is performed using a load-aware heuristic known as *LLFFD (Least-Loaded First-Fit Decreasing)*. During scheduling, the algorithm processes all incoming tasks sequentially, in their order of arrival. For each task $\tau_{i}$ , it iterates through the list of virtual machines $V=\left\{v_{1},v_{2},\ldots ,v_{n}\right\}$ and assigns $\tau_{i}$ to the VM $v_{j}$ with the lowest current CPU utilization $u_{j}$ that is capable of executing the task in a timely manner. The search follows a fixed order of VMs.

This heuristic mimics a classical load-balancing approach, where tasks are treated as incoming loads and VMs represent real-time execution slots with varying processing capabilities. By always assigning a new task to the least-loaded VM at the moment, LLFFD aims to balance the load among VMs, reduce idle resource time, and minimize task completion time. The procedure is formally defined as follows:

In the baseline scheduling strategy, task allocation is performed using a load-aware heuristic known as LLFFD (Least-Loaded First-Fit Decreasing). During scheduling, the algorithm processes all incoming tasks sequentially, in their order of arrival. For each task, it iterates through the list of virtual machines and assigns the task to the VM with the lowest current CPU utilization that is capable of executing the task in a timely manner. The search follows a fixed order of VMs. If all available VMs are already operating beyond a predefined utilization threshold, the task must wait in a queue until resources become available.

This heuristic mimics a classical load-balancing approach, where tasks are treated as incoming loads and VMs represent real-time execution slots with varying processing capabilities. By always assigning a new task to the least-loaded VM at the moment, LLFFD aims to balance the load among VMs, reduce idle resource time, and minimize task completion time. The procedure is formally defined as Equations 1–6:

For each task $\tau_{i}$ , virtual machines are assigned in the order of arrival $a\left(\tau_{1}\right)\leq a\left(\tau_{2}\right)\leq \cdots \leq a\left(\tau_{N}\right)$ :

$$v^{*}=\mathrm{arg min}U_{j}\left(t\right)$$ (1)

where v* represents the target VM, $\mathrm{arg min}U_{j}\left(t\right)$ represents the virtual machine with the lowest CPU utilization at time t.

#### 2.1.2 Load update mechanism

The computational complexity of LLFFD arises from checking the load of all VMs (V) for each of the tasks (T) , resulting in a time complexity of O(T×V) . However, This can be optimized to O(T⁡logV) by employing a priority queue (e.g., a min-heap) to maintain and retrieve the VM with the lowest load efficiently. The optimized process is defined as follows:

After allocation, update the load of the virtual machine:

$$U_{v^{*}}\left(t\right)\leftarrow U_{v^{*}}\left(t\right)+\frac{d\left(\tau_{i}\right)}{c\left(v^{*}\right)}$$ (2)

#### 2.1.3 Time complexity

It is important to note that LLFFD is inherently a greedy strategy—it makes allocation decisions based solely on the current load information, without any form of backtracking or global optimization. Once a task is allocated to a VM deemed optimal at that moment, the algorithm does not reconsider or adjust the assignments of previously scheduled tasks. This can lead to suboptimal results. For instance, a sequence of small tasks may overfill a relatively slow VM, while a faster VM remains underutilized due to a transiently higher load. Consequently, the overall system performance, especially task makespan, may degrade. Since LLFFD does not explore the global task-to-VM mapping space, there remains potential for improvement under specific workload distributions, as defined in:

1. **Basic implementation**: O(N×M) (Traverse all tasks and virtual machines)
2. **Heap optimization**: Maintain a min-heap to store virtual machine loads. The complexity of a single query is O(log⁡M) , and the total complexity is O(N⁡log⁡M)

### 2.2 Strategy 2- hybridization DE (FFDDE)

The proposed strategy is a hybrid algorithm that integrates the FFD heuristic into the evolutionary loop of Differential Evolution (DE). In our approach, a candidate solution to the scheduling problem is represented as a mapping of tasks to virtual machines (VMs), encoded as an integer vector of length N, where each element indicates the index of the VM assigned to a given task. For N tasks and M VMs, each candidate thus defines a complete assignment. Since standard DE operates on real-valued vectors, we adapt it to this discrete scheduling context by allowing mutation and crossover operators to generate intermediate (fractional) values, which are subsequently rounded or interpreted as discrete VM indices. To maintain solution feasibility—ensuring that no VM exceeds its task-handling capacity—we introduce a repair mechanism. If a VM ends up overloaded, we redistribute some of its tasks to less-loaded VMs using a strategy inspired by FFD, thereby preserving load balance.

The integration of FFD into DE occurs at two key stages:

(a) Population Initialization: The initial population of the DE algorithm is seeded with solutions generated using the FFD heuristic and some perturbed variants, providing DE with a strong starting point derived from domain-specific knowledge. (b) Heuristic Correction: After standard DE mutation and crossover, each newly generated candidate solution undergoes a heuristic refinement step. In this step, tasks are first sorted by length, and then reassigned to VMs using a first-fit approach that takes current VM loads into account. This leverages the FFD’s strength in fast and efficient task packing to guide the search process.

The fitness function for DE is defined as a weighted combination of total energy consumption and task makespan. Specifically, the fitness is calculated as:

Fitness = Total Energy Consumption+ λ× Makespan.

Where total energy consumption is the sum of energy used by all edge servers during scheduling, and makespan refers to the completion time of the last task. The weight λ is a positive constant introduced to penalize excessively long schedules, thereby encouraging energy-efficient solutions that also avoid severe task delays. This design favors strategies that consolidate tasks onto fewer servers—allowing others to remain idle or enter a low-power state—without causing unacceptable queuing delays on individual VMs.

The DE algorithm’s parameters were determined through a sensitivity analysis to ensure robust performance. The final configuration uses a population size of 50, a mutation factor F = 0.5, and a crossover rate CR = 0.9, as this combination was found to consistently provide an effective balance between convergence speed and solution quality in our experimental scenarios. The algorithm is executed for 100 generations or until the improvement in fitness falls below a predefined threshold.

By combining the global search capability of DE with the domain-specific efficiency of FFD, the hybrid algorithm aims to discover superior scheduling solutions compared to either method alone. It is worth noting that hybrid metaheuristics have shown strong potential in cloud and edge scheduling. For example, Yousif et al. (2024) demonstrated that seeding DE with heuristically generated solutions improved stability and execution time in IoT-edge task scheduling when compared with alternatives such as the Firefly Algorithm or Particle Swarm Optimization (PSO).

The definitations and descriptions are as follows:

#### 2.2.1 Encoding and initialization

**- Solution Vector**: $X=\left(x_{1},x_{2},\ldots ,x_{N}\right)$ , where $x_{i}\in \left\{1,2,\ldots ,M\right\}$ represents the ID of the virtual machine assigned to $\tau_{i}$ .

**- Initialize Population**: FFD solution, perturbed solution, random solution.

**- Fitness Function (Minimization Objective)**:

$$f\left(X\right)=\underset{\text{Total Energy Consumption}}{\underbrace{\overset{M}{\underset{j=1}{\sum }}\left(P_{j}\cdot T_{j}\right)}}+\lambda \cdot \underset{\text{Makespan}}{\underbrace{\underset{i\in N}{\max}C\left(\tau_{i}\right)}}$$ (3)

where $P_{j}$ refers to the power consumption of $VM_{j}$ , and $T_{j}$ corresponds to its runing time. λ=0.1 is the delay penalty coefficient.

#### 2.2.2 Discrete DE operations (based on standard DE improvement)

**- Mutation (for individual** $X_{k}$ **)**:

$$v_{ki}=\text{round}\left(x_{\mathrm{r1i}}+F\cdot \left(x_{\mathrm{r2i}}-x_{\mathrm{r3i}}\right)\right),\;F=0.5$$ (4)

where k denotes the index of the current individual being processed $r_{1},r_{2},r_{3}$ are distinct random indices, and $x_{r_{i}j}$ denotes the i -th component of the random individual.

**- Crossover (Generating Vector** $U_{k}$ **)**:

$$u_{ki}=\left\{\begin{array}{cc} v_{ki},\; & \text{if rand}\left(\right)\leq CR\text{ or }i=i_{\text{rand}}\\ x_{ki},\; & \text{otherwise} \end{array}\right.,\;CR=0.9$$ (5)

$i_{\text{rand}}$ is a random dimension index, ensuring at least one dimension comes from the mutation vector.

#### 2.2.3 FFD hybrid mechanism

**- Repair Population**: 50% of individuals are derived from $X_{\text{FFD}}$ (FFD-based baseline solution) and its perturbations (randomly swap 10% of task assignments); the rest are random solutions.

**- Feasibility Repair Operator**: For an overloaded virtual machine $v_{j}$ , reorder its tasks by $d\left(\tau_{i}\right)$ randomly, then reassign them sequentially to the virtual machine with the lowest load:

$$v_{\text{new}}^{*}=\arg\underset{v_{q}\in V}{\min}U_{q}\left(t\right)$$ (6)

Update $U_{v^{*}}\left(t\right)\leftarrow U_{v^{*}}\left(t\right)+d\left(\tau_{i}\right)/c\left(v*\right)$ (consistent with FFD load calculation).

- algorithm steps are as follows:

Algorithm 1 Proposed Evolutionary Algorithm.

**Input**: Task set T , virtual machine set V , maximum number of generations $G_{\text{max}}=100$ , fitness threshold $\varepsilon =10^{-4}$

**Output**: Optimal solution $x^{*}$

1: Initialize population $P_{0}=\left\{\text{FFD solution},\text{perturbed solution},\text{random solution}\right\}$

2: **for** g=1 **to** $G_{\text{max}}$ **do**
3:   **for** each $X_{k}\in P_{g}$ **do**
4:    Generate mutation vector $v_{k}$

5:    Generate trial vector $u_{k}$

6:    Perform FFD repair on $u_{k}$

7:    **if** $u_{k}$ contains overloaded VMs **then**
8:      **for** each overloaded VM $v_{j}$ in $u_{k}$ **do**
9: $T_{j}\leftarrow$ Identify all tasks currently assigned to $v_{j}$ .
10:        Sort tasks in $T_{j}$ in non-increasing order of computational demand.
11:        **for** each task $t_{i}$ in sorted $T_{j}$ **do**
12:         Find the VM $v_{\mathrm{best}}$ with the lowest current load.
13:         Reassign task $t_{i}$ to $v_{\mathrm{best}}$ and update loads.
14:        **end for**
15:      **end for**
16:    **end if**
17:    Calculate $f\left(u_{k}\right)$

18:    **if** $f\left(u_{k}\right)\leq f\left(x_{k}\right)$ **then**, $P_{\left\{g+1\right\}}\leftarrow u_{k}$

19:    **end if**
20:   **end for**
21:   **if** $\max|f\left(X_{\left\{k,g\right\}}\right)-f\left(X_{\left\{k,g-1\right\}}\right)|<\varepsilon$ **then**, Terminate early
22:   **end if**
23: **end for**

**FIGURE 2:**
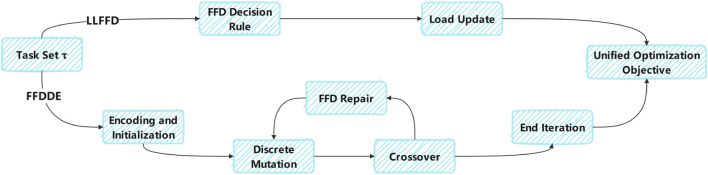
Comparison of processes between LLFFD and FFDDE.

- The comparison of processes between LLFFD and FFDDE are shown in Figure 2 as follows:

#### 2.2.4 Time complexity

The overall time complexity of the FFDDE algorithm is (G.P. (NlogN + NlogM)), where G is the maximum number of generations, P is the population size, N is the total number of tasks, and M is the number of virtual machines. This complexity primarily stems from the algorithm’s core iterative loop: for each individual (P) in each generation (G), the algorithm needs to perform an FFD repair operation. This operation, which includes sorting the tasks (O(NlogN)) and reassigning them to the virtual machine with the lowest load (O(NlogM)), is the most computationally expensive part of the process. Since G and P are typically predefined constants, the algorithm’s execution efficiency is mainly influenced by the number of tasks and virtual machines.

## 3 Simulation environment

Conducting large-scale experiments in real-world edge computing environments remains a significant challenge. As a result, many studies rely on simulation platforms to evaluate the performance of energy-efficient scheduling algorithms. EdgeCloudSim is a leading Java-based simulation framework capable of modeling the interactions between mobile devices, edge nodes, and network topologies. This work focuses on the “end device–edge node” architecture within EdgeCloudSim, leveraging a dynamic energy-aware scheduler to coordinate heterogeneous resources and jointly optimize task execution efficiency and energy consumption. The core components of this architecture are as follows:

Terminal device layer (Task Generator Layer).

Mobile devices or sensors generate constrained computational tasks characterized by parameters such as data volume, deadlines, and dependencies. These tasks are modeled using Directed Acyclic Graphs (DAGs) to capture task topologies. A local decision module estimates the energy and latency of local execution, providing a baseline for offloading decisions.

**Edge computing layer**, which includes:

- Task scheduler, whose core purpose is to optimize energy consumption under load balancing.
- Edge hosts, each edge host is physically present and generates virtual machines to complete tasks. The energy consumption of VMs is reflected through edge hosts.

**FIGURE 3:**
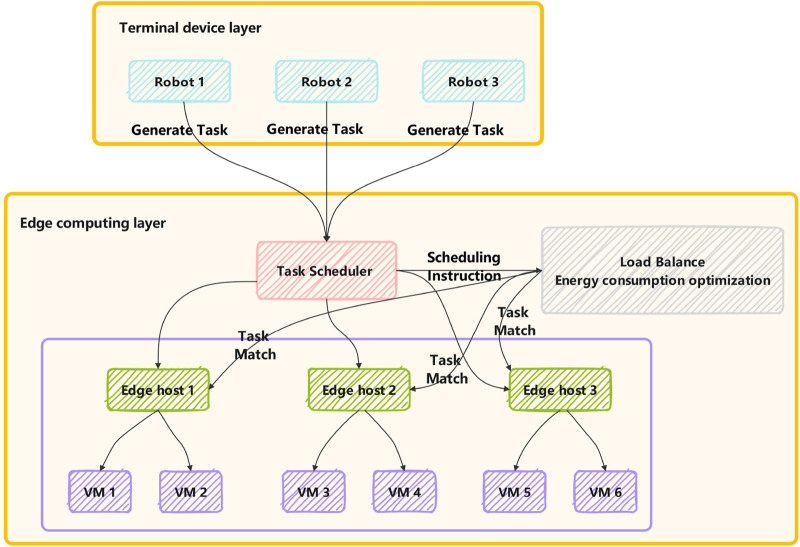
Structure of edge computing simulation environment.

Furthermore, EdgeCloudSim integrates four representative task types to reflect the computational demands across diverse edge computing scenarios:

AUGMENTED_REALITY: Simulates high-throughput tasks involving image processing and rendering.
HEALTH_APP: Represents real-time physiological signal monitoring and analysis, with high sensitivity to latency and reliability.
HEAVY_COMP_APP: Captures compute-intensive applications such as robotic path planning or complex model inference.
INFOTAINMENT_APP: Encompasses video streaming and content distribution, requiring moderate bandwidth and stability.
The overall system workflow is illustrated in Figure 3.

## 4 Experiments

### 4.1 Experimental setup

In this study, we constructed an edge computing simulation environment using the EdgeCloudSim platform to evaluate the performance differences between the proposed hybrid Differential Evolution (DE) algorithm—incorporating traditional control heuristics—and the baseline LLFFD heuristic scheduling strategy. The experimental environment consists of a small-scale edge computing cluster comprising four physical edge servers, each configured with three virtual machines (VMs), totaling 12 VMs in the simulation.

Two task groups were simulated, with the primary focus on a lightweight workload composed of 160 tasks. These tasks were evenly distributed across four representative edge computing application categories, with 40 tasks per category:

Augmented Reality Applications (AUGMENTED_REALITY): Represent tasks related to image processing and rendering with high throughput demands. These tasks characterize compute-intensive requirements typical of edge-based augmented reality scenarios, such as object recognition or real-time scene rendering.

Health Monitoring Applications (HEALTH_APP): Encompass real-time physiological monitoring and analytical tasks. These are highly sensitive to latency and require high reliability, reflecting real-time analysis and alerting services in healthcare contexts.

Heavy Computational Applications (HEAVY_COMP_APP): Include tasks such as robotic path planning and complex model inference, which demand significant computational resources. These tasks are typically less sensitive to short-term latency fluctuations.

Infotainment Applications (INFOTAINMENT_APP): Comprise video streaming and content distribution workloads with moderate bandwidth requirements and a certain level of stability. These tasks are representative of mobile video services and media content delivery at the network edge. During simulation, all tasks are injected into the system at approximately the same time (around the 200-s mark), emulating a high-concurrency task submission scenario. This experimental configuration is designed to intensify the scheduling challenge and provide a rigorous environment for performance comparison. Under such conditions, the resource management capabilities of the two scheduling approaches—particularly in terms of task execution latency, energy consumption, and resource allocation efficiency—can be effectively evaluated and contrasted. The specific parameters are as follows:

1. Task end: To create a realistic and diverse workload, the characteristics of the 160 tasks were defined based on their application category. The computational demand and data size for each task type followed a normal distribution, with parameters detailed in Table 2.
2. Computing end: The simulated edge environment consists of 12 virtual machines with heterogeneous capabilities, as detailed in Table 3.

**TABLE 2:** Task set.

Application category	Number of tasks	Computational demand
AUGMENTED REALITY	40	2000 MI
HEALTH APP	40	1250 MI
HEAVY COMP APP	40	3000 MI
INFOTAINMENT APP	40	2000 MI

**TABLE 3:** VM set.

VM 1	VM 2	VM 3	VM 4	VM 5	VM 6
1,100 (MIPS)	2,500 (MIPS)	1,600 (MIPS)	1,400 (MIPS)	2000 (MIPS)	2000 (MIPS)

VM 7	VM 8	VM 9	VM 10	VM 11	VM 12
3,000 (MIPS)	1,000 (MIPS)	2,900 (MIPS)	1,000 (MIPS)	1800 (MIPS)	1,300 (MIPS)

### 4.2 Experimental results

To ensure the statistical robustness of the research results and mitigate the impact of random fluctuations, we independently repeated each set of simulation experiments for both FFDDE and GA (population size = 50, F = 0.5, and CR = 0.9) strategies 30 times. Note that the results of each LLFFD run were the same.

**FIGURE 4:**
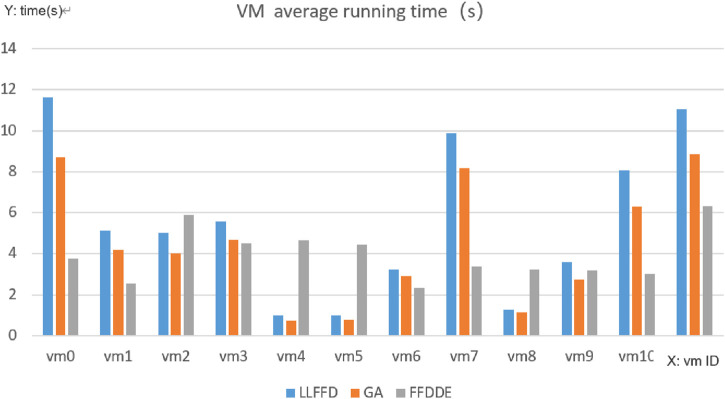
Comparison of average running time.

As illustrated in Figure 4, regarding the average task execution time, the traditional LLFFD algorithm recorded 5.86 s (standard deviation ± 3.85s). After incorporating the Genetic Algorithm (GA), the average time decreased to 4.43 s (SD ± 2.91s), and the FFDDE algorithm further reduced it to 3.95 s (SD ± 1.15s). Compared to LLFFD, GA and FFDDE shortened the average task execution time by approximately 24% and 33%, respectively. Moreover, FFDDE had the smallest standard deviation, indicating a significant reduction in the volatility of its task execution times and demonstrating a clear advantage in minimizing both task waiting and execution durations.

**FIGURE 5:**
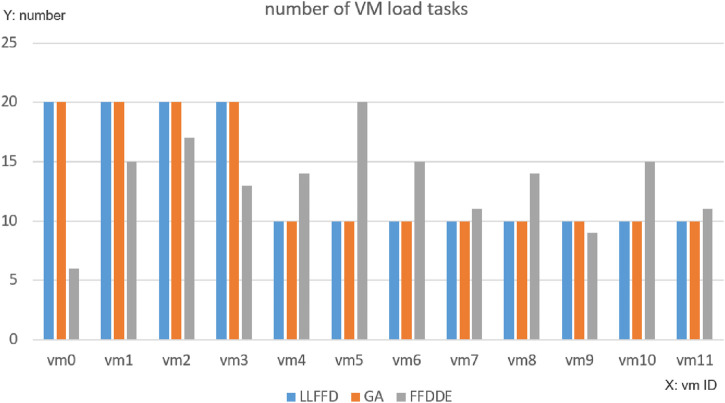
Comparison of VM loads.

As shown in Figure 5, under the traditional LLFFD strategy, tasks were significantly concentrated on a limited subset of virtual machines, with several VMs operating at full capacity (four in the case of LLFFD, compared to only one under FFDDE and GA). In contrast, FFDDE and GA, the heuristic algorithms exhibited a more balanced load distribution across VMs. This indicates that LLFFD tends to produce load imbalance, leading to the formation of performance hotspots on specific virtual machines, which in turn results in noticeable task execution delays and increased energy consumption.

**FIGURE 6:**
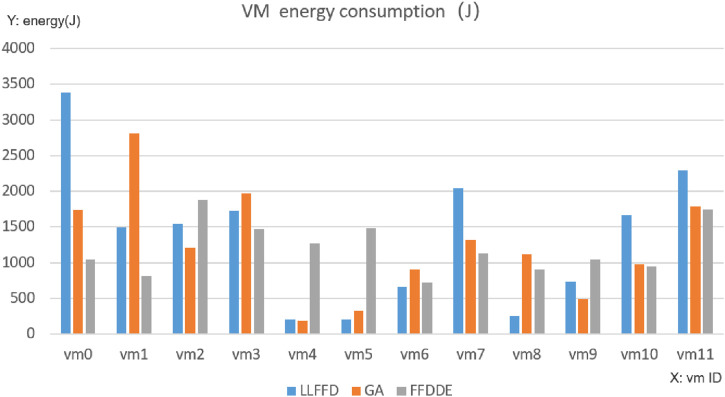
Comparison of energy consumpiton.

As illustrated in Figure 6, the three algorithms demonstrated distinct differences in total energy consumption. The traditional LLFFD algorithm consumed the most energy, totaling 16,189 J. In comparison, the GA algorithm used 14,809 J, while the FFDDE algorithm was the most efficient, consuming only 14,429 J. This translates to energy savings of approximately 8.5% for GA and a more significant 10.9% for FFDDE, relative to the LLFFD baseline. A further analysis of per-task energy consumption highlights the superiority of FFDDE. It reduced the average energy per task from 101.2 J (for LLFFD) to 90.2 J, while also lowering the standard deviation from 79.7 to 70.7. This indicates that the FFDDE algorithm utilizes computing resources more efficiently, thereby effectively mitigating high energy consumption peaks.

Virtual Machine Load Distribution Performance.

There are significant differences in the task virtual machine allocation methods among the three algorithms: The FFDDE algorithm achieves better load balancing, more even task distribution, and a more reasonable number of tasks executed by each virtual machine (ranging from 6 to 20 tasks), without obvious hotspots or resource idle situations. Correspondingly, the difference in average execution time and average energy consumption of each virtual machine has significantly reduced.

## 5 Conclusions and future work

In this study, we addressed the challenge of energy-efficient task scheduling in resource-constrained edge computing environments by developing a novel hybrid algorithm (FFDDE) that combines a traditional heuristic scheduler with a Differential Evolution (DE) metaheuristic. The proposed FFDDE approach builds on the conventional FFD heuristic algorithm but augments it with evolutionary optimization to refine scheduling decisions. We implemented and evaluated FFDDE in a simulated edge environment using EdgeCloudSim, which provides realistic modeling of both computational and network constraint. Our experimental results demonstrate that the hybrid FFDDE algorithm significantly outperforms the traditional LLFFD heuristic in terms of energy efficiency, execution time, and load balancing. In particular, FFDDE achieved lower overall energy consumption and reduced the average task execution time compared to LLFFD. At the same time, it distributed workloads more evenly across available edge servers, avoiding the severe load imbalances observed under the baseline heuristic. These improvements confirm that combining heuristic rules with evolutionary search enables more effective scheduling under the strict resource limitations of edge computing. By leveraging the fast decision-making of LLFFD and the global optimization capability of DE, the hybrid FFDDE approach was able to navigate trade-offs between energy usage and performance, yielding superior outcomes in the EdgeCloudSim-based simulations. The findings underscore that a hybrid heuristic–evolution strategy can better satisfy the competing demands of energy efficiency and latency in edge environments than heuristic methods alone.

Looking ahead, this research has several important directions for future work. First, we plan to deploy the FFDDE scheduler on a real-world edge computing testbed to validate its effectiveness under actual network variability and hardware heterogeneity. The research will also be extended to accommodate dynamic and unpredictable workloads, enabling the scheduler to respond in real-time to changes in task patterns and resource availability. Furthermore, to achieve more comprehensive sustainability goals, we will explore new area that consider a task’s carbon emissions and heat dissipation requirements to enable “greener” computing. At the same time, through smarter scheduling strategies, we will also investigate how to extend the lifespan of edge devices and reduce e-waste, thereby implementing resource recycling and hardware lifecycle management. Finally, we can explore integrating other metaheuristic algorithms into a hybrid framework to achieve multi-objective optimization, balancing energy consumption, latency, and other quality-of-service metrics simultaneously.

## Data Availability

The raw data supporting the conclusions of this article will be made available by the authors, without undue reservation.
